# Editorial: Emotional impulsivity and emotion regulation deficits as important factors in clinically challenging behaviors in psychiatric disorders

**DOI:** 10.3389/fpsyt.2025.1595135

**Published:** 2025-04-01

**Authors:** Matthew J. Hoptman, Melissa A. Cyders, Anthony O. Ahmed

**Affiliations:** ^1^ Clinical Research Division, Nathan S. Kline Institute for Psychiatric Research, Orangeburg, NY, United States; ^2^ Department of Psychiatry, NYU Grossman School of Medicine, New York, NY, United States; ^3^ Department of Psychology, Indiana University Indianapolis, Indianapolis, IN, United States; ^4^ Department of Psychiatry, Weill Cornell Medicine, New York, NY, United States

**Keywords:** urgency, impulsivity, emotion regulation, problem behaviors, psychopathology

In the Research Topic “*Emotional Impulsivity and Emotion Regulation Deficits as Important Factors in Clinically Challenging Behaviors in Psychiatric Disorders*”, we examine the role of emotion-related impulsivity (also known as “urgency”) and emotion regulation deficits in behaviors such as suicidality, nonsuicidal self-injury, pathological eating, and mood disorders. The articles in this Research Topic use a range of methodologies, including ecological momentary assessment, experimental paradigms, self-report methods, and neuroimaging to study urgency and its correlates. In addition, the articles provide accounts of how urgency may play out in the context of broader theories of emotion and nonemotion-based models. This Research Topic thus represents a comprehensive examination of urgency and emotion dysregulation and how they interact with each other and relate to psychopathology. We are fortunate to have received contributions of more than 10 articles from leaders in the field.

Prior work had established urgency and emotion dysregulation as important factors in psychopathology and challenging clinical behaviors (1). The studies in this Research Topic support and extend this prior work. For example, in a preliminary study, Hoptman et al. used an fMRI emotion regulation task to show that people with schizophrenia who have high levels of suicidal ideation and behavior (SIB) have reduced activation in emotion regulation-relevant circuitry compared to those with lower levels of SIB in multiple regions, including medial prefrontal cortex, rostral anterior cingulate, superior frontal gyrus, dorsolateral prefrontal cortex, right middle cingulate, and right superior temporal gyrus. Moreover, across groups, higher activation in right middle cingulate gyrus, right superior temporal gyrus, and right DLPFC correlated with lower levels of negative urgency. This finding suggests that deficient activation in these regions might be associated with the kinds of emotion dysregulation seen in suicidal ideation and behavior. Prior work with this sample showed that people in the high SIB group had highly elevated negative urgency compared to those in the low SIB group (2). Consistent with this finding, Ortin-Peralta et al. identified pathways between negative and positive urgency and intergenerational transmission of suicide risk in children. In an in-depth examination of the interpersonal theory of suicide, Ranjbar et al. showed that emotion dysregulation mediated the pathways between suicidal ideation constructs, such as perceived burdensomeness and thwarted belongingness on one hand and suicidal behavior on the other. In healthy adolescents, Fisher-Fox et al. found that baseline urgency predicted increased emotion dysregulation over time, providing support for a temporal relationship between the two constructs.

Two articles in those exhibiting non-suicidal self-injury (NSSI) highlighted the importance of emotion dysregulation in this phenomenon. Thus, Liu et al. showed that lower levels of self-compassion and lower usage of cognitive reassessment emotion regulation strategies were associated with higher levels of NSSI in adolescents with mood disorders. Along similar lines, Ge et al. showed that relationships between a profile of neuroticism, childhood victimization, poor resilience, and family dysfunction on one hand and NSSI on the other was mediated by emotion dysregulation, which was in turn moderated by maladaptive cognitive emotion regulation. Finally, Belloli et al. identified links between emotional dysregulation and psychopathological traits such as anxiety and depression in candidates for bariatric surgery. In each of these cases, the work described the critical role of urgency and/or emotion dysregulation in these clinically challenging behaviors.

Two articles examine the role of urgency in broader theories of emotion Fisher-Fox et al.) and in contexts beyond emotion. Elliott et al. Fisher-Fox et al. consider ways in which to integrate urgency into broader existing emotion theories that highlight adaptive, in addition to maladaptive, impacts of emotions on behavior, as a means to catalyze future research in this area. Elliott et al. tested whether emotions uniquely lead to urgency, finding that while emotions are key in driving risk-taking, people high in urgency may demonstrate risk-taking in other physiologically charged contexts as well (e.g., tiredness and hunger).

Finally, two articles represented methodological advances. Ajilore et al. examined entropy of passive phone keystrokes and found that it was associated with performance on tasks of executive function and correlated with both depressive symptoms and the variability of phone app-derived impulsive feelings in people with bipolar disorder. Finally, Allen et al. used an innovative task that examined emotional and nonemotional working memory, as well as affective flexibility. They found that neutral, but not emotional, working memory performance correlated with emotion-related impulsivity and internalizing psychopathology. By linking cognitive performance with emotion and impulsivity, the results of both articles imply shared or overlapping mechanisms of cognitive performance and the regulation of emotion and action, as well as the likely centrality of cognitive control.

The articles in this Research Topic highlight and extend the importance of urgency in psychopathology. Further work will help identify the distinctions between urgency and emotion dysregulation and will provide important clues to how emotion-related impulsivity influences emotion dysregulation and thereby may lead to dysregulated behavior. Future directions likely will include using neuroimaging to examine the neural correlates of these constructs, experimental paradigms to better establish causality and mechanisms, and ecological momentary assessment to better understand the complex and reciprocal interactions between urgency and emotion dysregulation over time. A better understanding of the phenomenology, neural circuitry, and temporal nature of urgency and emotional dysregulation is likely to have treatment implications, whether by psychological, neuromodulatory, pharmacological, or neurofeedback mechanisms. This knowledge will likely lead to improved treatment approaches to address challenging behaviors in numerous clinical populations.
